# MEF2C regulates osteoclastogenesis and pathologic bone resorption via c-FOS

**DOI:** 10.1038/s41413-020-00120-2

**Published:** 2021-01-11

**Authors:** Takayuki Fujii, Koichi Murata, Se-Hwan Mun, Seyeon Bae, Ye Ji Lee, Tannia Pannellini, Kyuho Kang, David Oliver, Kyung-Hyun Park-Min, Lionel B. Ivashkiv

**Affiliations:** 1 Arthritis and Tissue Degeneration Program and David Z. Rosensweig Genomics Research Center, Hospital for Special Surgery, New York, NY 10021 USA; 2 Department of Orthopaedic Surgery, Kyoto University Graduate School of Medicine, Sakyo, Kyoto, 606-8507 Japan; 3 Department of Advanced Medicine for Rheumatic Diseases, Kyoto University Graduate School of Medicine, Sakyo, Kyoto, 606-8507 Japan; 4 Department of Biology, Chungbuk National University, Cheongju, 28644 Republic of Korea; 5 BCMB Allied Program, Weill Cornell Graduate School of Medical Science, New York, NY 10021 USA; 6 Immunology and Microbial Pathogenesis Program, Weill Cornell Graduate School of Medical Science, New York, NY 10021 USA

**Keywords:** Bone, Homeostasis

## Abstract

Osteoporosis is a metabolic bone disease with dysregulated coupling between bone resorption and bone formation, which results in decreased bone mineral density. The *MEF2C* locus, which encodes the transcription factor MADS box transcription enhancer factor 2, polypeptide C (MEF2C), is strongly associated with adult osteoporosis and osteoporotic fractures. Although the role of MEF2C in bone and cartilage formation by osteoblasts, osteocytes, and chondrocytes has been studied, the role of MEF2C in osteoclasts, which mediate bone resorption, remains unclear. In this study, we identified MEF2C as a positive regulator of human and mouse osteoclast differentiation. While decreased MEF2C expression resulted in diminished osteoclastogenesis, ectopic expression of MEF2C enhanced osteoclast generation. Using transcriptomic and bioinformatic approaches, we found that MEF2C promotes the RANKL-mediated induction of the transcription factors c-FOS and NFATc1, which play a key role in osteoclastogenesis. Mechanistically, MEF2C binds to *FOS* regulatory regions to induce c-FOS expression, leading to the activation of *NFATC1* and downstream osteoclastogenesis. Inducible deletion of *Mef2c* in mice resulted in increased bone mass under physiological conditions and protected mice from bone erosion by diminishing osteoclast formation in K/BxN serum induced arthritis, a murine model of inflammatory arthritis. Our findings reveal direct regulation of osteoclasts by MEF2C, thus adding osteoclasts as a cell type in which altered MEF2C expression or function can contribute to pathological bone remodeling.

## Introduction

Osteoclasts are myeloid lineage cells that resorb bone.^1,2^ As bone constantly remodels, remodeling is tightly regulated by osteoclasts, bone-forming osteoblasts, and osteocytes.^3^ The balance between bone resorption and formation is critically important for the maintenance of skeletal integrity and disruption of this balance results in pathological changes in bone structure and quality. Overly active osteoclasts lead to an imbalance, tipping the scale toward more bone resorption than formation and leading to pathological bone loss, such as occurs in patients with osteoporosis.^4,5^ Extensive efforts have been made to identify the risk factors associated with osteoporosis and osteoporotic bone fractures and understand the pathogenesis of pathological bone loss. During the last decade, genome-wide association studies (GWASs) assaying hundreds of thousands of single nucleotide polymorphisms (SNPs) have identified genetic variants that are associated with osteoporosis and related traits.^6–8^

Recent studies show that SNPs associated with the *MEF2C* locus are linked to adult osteoporosis and osteoporotic fractures.^6–8^ MEF2C is a transcription factor known to be involved in the development of a variety of cells, including muscle, neural, chondroid, immune, and endothelial cells.^9–13^ MEF2C function is regulated by interaction with other transcription factors and co-activators, and MEF2C undergoes extensive posttranslational modification. *Mef2c* germline deletion in mice leads to embryo hypoplasia, disorganized myofibers, and death at embryonic day 9.5 from cardiovascular development defects.^9,14^ *Mef2c* heterozygous null mice reach birth but display severe deficiency in ossification of the sternum at postnatal day 1, implying an important role of MEF2C in bone development.^11^ MEF2C and MEF2D double deficiency in endochondral cartilage results in impaired hypertrophy, cartilage angiogenesis, ossification, and longitudinal bone growth in mice. Furthermore, genetic deletion of *Mef2c* in osteocytes has been shown to increase bone mass due to decreased expression of sclerostin (encoded by *SOST*), a potent inhibitor of canonical WNT signaling pathways.^15,16^ MEF2C deletion in a murine osteoblastic cell line also causes decreased RUNX2 expression and bone mineralization.^17^ The studies mentioned have established MEF2C as a significant player in osteoblast function and bone formation. However, the direct role of MEF2C in osteoclast differentiation and function has largely been overlooked.

RANKL is a TNF family cytokine that is a key driver of osteoclastogenesis and signaling pathways downstream of its receptor RANK are well-established.^18^ During osteoclastogenesis, RANKL induces many transcription factors including MYC, NF-κB, c-FOS, and NFATc1, which work in a complex network to promote osteoclast differentiation.^19–22^ c-FOS is a basic leucine zipper transcription factor that forms heterodimers with c-JUN to form AP-1 complexes. c-FOS is induced by various stimuli including mitogen-activated protein kinase (MAPK) signal transduction pathways and the Ca^2+^/calmodulin-dependent kinase (CaMK)-CREB pathway.^23^ However, how c-FOS expression is regulated after RANKL stimulation is not completely understood.

In this study, we used human osteoclast precursor cells (OCPs) to identify the role of MEF2C in osteoclastogenesis. We examined whether MEF2C is associated with the function or generation of osteoclasts. In particular, we showed that MEF2C is a positive regulator of osteoclastogenesis in both human and mouse OCPs by interacting with *FOS* upstream regulatory regions. We also show that deletion of *Mef2c* at 6 weeks of age in mice increases bone mass by diminishing osteoclast numbers without altering osteoblast number and function. *Mef2c* inducible deletion protected mice from arthritic bone erosion by suppressing osteoclasts. Our results suggest the importance of a MEF2C/c-FOS axis in osteoclastogenesis and provide mechanistic insight into how MEF2C is associated with bone metabolism in humans.

## Results

### MEF2C is a positive regulator of human osteoclastogenesis

**Fig. 1:**
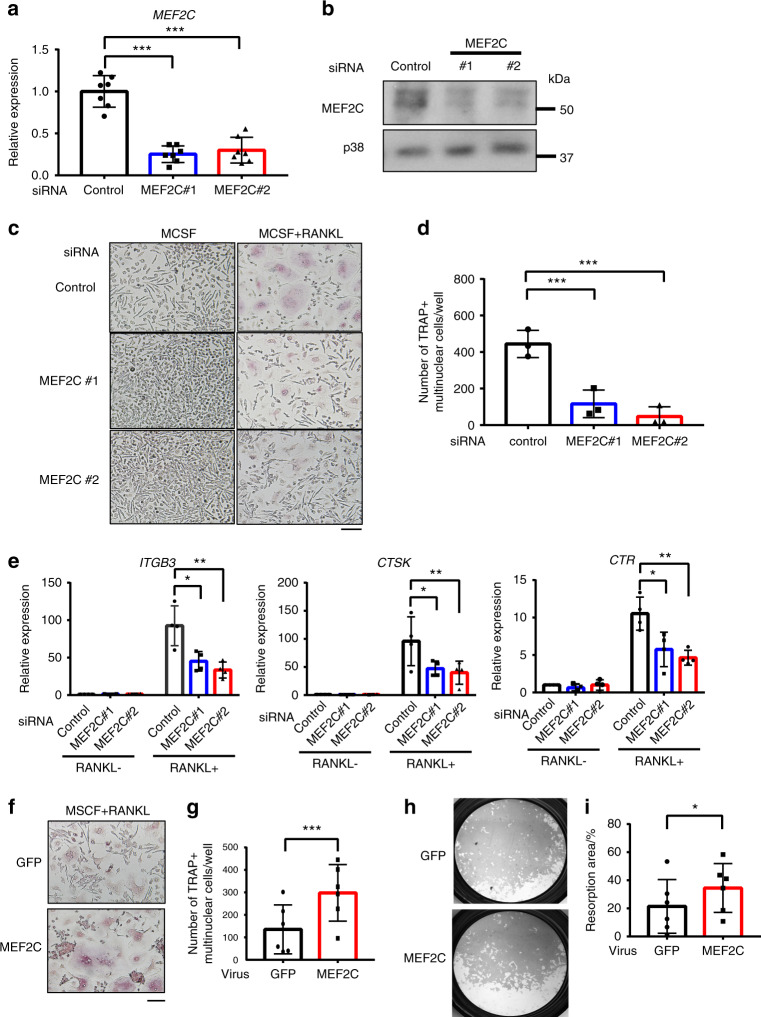
MEF2C is a positive regulator of human osteoclastogenesis. **a–e** Human osteoclast precursor cells were nucleofected with control or two different MEF2C siRNAs. **a, b** MEF2C mRNA and protein expression. **c, d** Osteoclastogenesis assay. **c** TRAP staining of human osteoclasts. Scale bar, 100 μm. **d** Osteoclast number of three independent experiments. **e** RT-qPCR analysis of *ITGB3*, *CTSK*, and *CTR* mRNA after 72 h of culture with or without RANKL (40 ng·mL^−1^) normalized relative to *TBP* mRNA. Control samples without RANKL were set at 1.0. (*n* = 3). **f–h** Human osteoclast precursor cells were transduced with adenoviral particles encoding GFP or MEF2C-FLAG. **f** TRAP staining of human osteoclasts transduced with GFP or MEF2C. Scale bar, 100 μm. **g** Cumulative data showing numbers of osteoclasts from six independent experiments. **h** Representative images of bone resorption assay of human osteoclasts transduced with GFP or MEF2C. **i** Quantitation of resorption area from six different experiments. Statistics used: **a**, **d** repeated measurement one-way ANOVA **e** repeated measurement Two-way ANOVA, **g**, **i** paired *t*-test. *P < 0.05, **P < 0.01, ***P < 0.001. Data were shown as mean ± SD

To identify the role of MEF2C in human osteoclast differentiation, we used human blood-derived osteoclast precursor cells (OCPs), which are relevant to human diseases.^24,25^ Although the expression of MEF2C mRNA and protein in OCPs was diminished by RANKL treatment during the first 2 days of osteoclast differentiation, the levels of MEF2C persisted during osteoclastogenesis (Supplementary Fig. S1a, b). To test the function of MEF2C in human osteoclastogenesis, MEF2C expression was knocked down by short interfering RNA (siRNA) and cells were differentiated into osteoclasts as previously described.^25^ We utilized two different siRNAs targeting MEF2C, and both MEF2C siRNAs effectively suppressed mRNA and protein expression of MEF2C in OCPs prior to RANKL stimulation (Fig. 1a, b, Supplementary Fig. S1c). Strikingly, decreased expression of MEF2C in OCPs resulted in diminished osteoclast differentiation after RANKL stimulation (Fig. 1c, d, Supplementary Fig. S1d). The expression of osteoclast marker genes such as Integrin Beta 3 (*ITGB3*), Cathepsin K (*CTSK*), and Calcitonin Receptor (*CTR*) were accordingly suppressed in MEF2C knockdown (KD) cells compared to control cells (Fig. 1e).

To obtain additional genetic evidence for a positive role of MEF2C in osteoclastogenesis, we transduced human OCPs with adenoviruses encoding FLAG-tagged MEF2C or GFP, as previously described.^25^ The expression of MEF2C mRNA and protein modestly increased in MEF2C-transduced cells under RANKL stimulation and was also detected in the nucleus by immunohistochemistry (IHC) analysis (Supplementary Fig. S1e–h). Ectopic expression of MEF2C enhanced RANKL-induced osteoclast differentiation and bone resorption (Fig. 1f–i). Accordingly, osteoclast marker genes showed increased expression in MEF2C-transduced cells compared to control GFP-transduced cells (Supplementary Fig. S1j). However, overexpression of MEF2C did not affect cell viability (Supplementary Fig. S1i). These results suggest that MEF2C is a positive regulator of human osteoclastogenesis.

### MEF2C regulates physiological bone remodeling

**Fig. 2:**
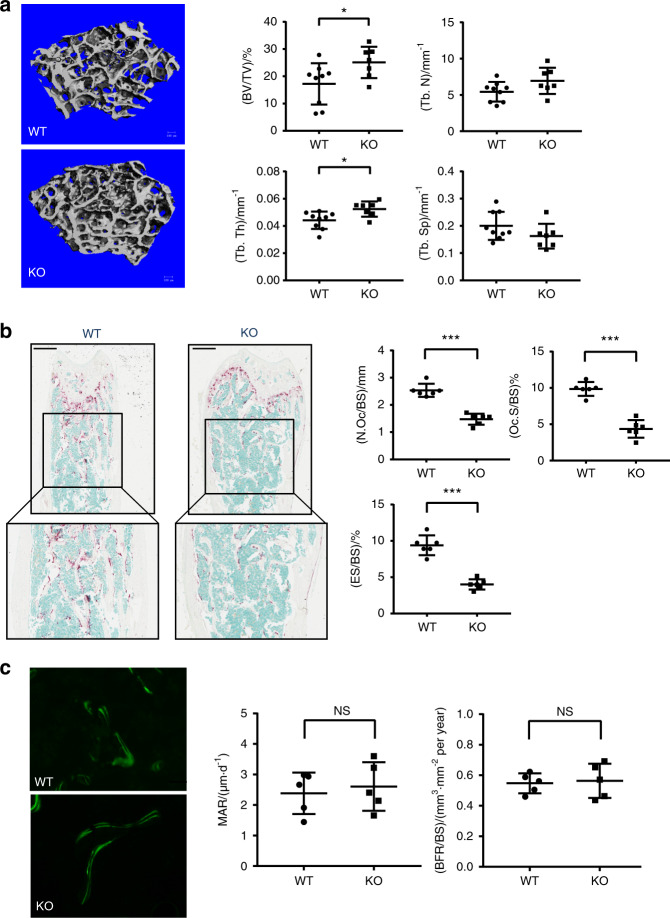
MEF2C^ΔMX^ mice show increased bone mass with decreased osteoclast numbers. **a** μCT analysis of femurs from 16-week-old male MEF2C^ΔMX^ KO (*n* = 7) and littermate control WT mice (*n* = 9). Right panels show the indicated parameters in distal femurs. Bone volume/tissue volume ratio (BV/TV), trabecular thickness (Tb.Th), trabecular numbers (Tb.N), and porosity were determined by μCT analysis. **b** Histomorphometry analysis of the distal femur of 16-week-old male mice. Representative images showing TRAP-positive, multinucleated osteoclasts (red). Scale bars, 500 μm. Right panels show number of osteoclasts per bone surface (N.Oc/BS), osteoclast surface area per bone surface (Oc.S/BS) and eroded surface per bone surface (ES/BS). **c** Dynamic bone histomorphometry analysis of the distal femur. Scale bars, 50 μm. Representative images showing casein incorporation into newly calcifying bone. Right panels showed mineral apposition rate (MAR) and bone formation rate (BFR/BS). Data are shown as mean ± SD. Statistics used: **a, b, c** Welch’s *t*-test. NS; not significant, *P < 0.05, **P < 0.01

We wanted to test whether MEF2C regulates in vivo osteoclastogenesis. MEF2C deficiency leads to embryo hypoplasia, disorganized myofibers, and perinatal lethality.^9,14^ To avoid any developmental defects caused by MEF2C deficiency, we generated MEF2C inducible conditional deficient mice (MEF2C^ΔMX^) by crossing MEF2C floxed mice with Mx1 cre mice, where deletion of the MEF2C gene was induced by the Mx1 promotor-driven Cre recombinase that was activated by injection of polyinosine: polycytidylic acid (poly I:C).^26^ To investigate the in vivo function of MEF2C in bone metabolism, MEF2C was deleted at the age of 6 weeks; MEF2C^ΔMX^ mice showed no overt physical abnormalities including body weight and femur length at 16 weeks of age (Supplementary Fig. S2a). To assess the efficiency of MEF2C deletion, we performed IHC staining of MEF2C in the distal femur. MEF2C was detected in many cells including bone marrow cells, osteocytes, and chondrocytes in femurs from both WT and MEF2C^ΔMX^ mice (Supplementary Fig. 2b). The percentage of MEF2C-positive cells was significantly diminished in bone marrow cells in MEF2C^ΔMX^ mice relative to WT mice while there was no significant difference in MEF2C-postive staining in chondrocytes and osteocytes between WT and MEF2C^ΔMX^ mice (Supplementary Fig. S2b). To identify which bone marrow cell populations are positive for MEF2C in WT and in which cell types MEF2C is deleted by Mx1-cre, we used fluorescence-activated cell sorting to purify T cells, B cells, neutrophils, monocytes, and OCPs from bone marrow cells of the femur and the tibia^27^ (gating strategy shown in Supplementary Fig. S2c), and then cells were stained with MEF2C antibodies. In WT cells, the expression of MEF2C was negligible in neutrophils and monocytes and was marginally positive for T cells and B cells. In contrast, MEF2C expression was high in sorted Ly6C^+^CD11b^med/low^ OCPs from WT mice and was remarkably reduced in OCPs from MEF2C^ΔMX^ mice (Supplementary Fig. S2d). These results suggest that this model led to the diminished expression of MEF2C in osteoclast precursor cells. In micro-CT analysis, 16-week-old MEF2C^ΔMX^ male mice exhibited increased bone mass, where BV/TV and Tb.Th were significantly increased compared to littermate control MX cre mice (WT) (Fig. 2a). Histomorphometric analysis revealed the number of osteoclasts, osteoclast surfaces, and eroded surfaces to be significantly lower in MEF2C^ΔMX^ mice than control mice (Fig. 2b). In contrast, there were no significant differences in the activity of osteoblasts in MEF2C^ΔMX^ mice compared to control mice (Fig. 2c). Therefore, our findings propose that MEF2C deficiency in adulthood results in increased bone mass due to decreases in the number of osteoclasts under physiological conditions.

**Fig. 3:**
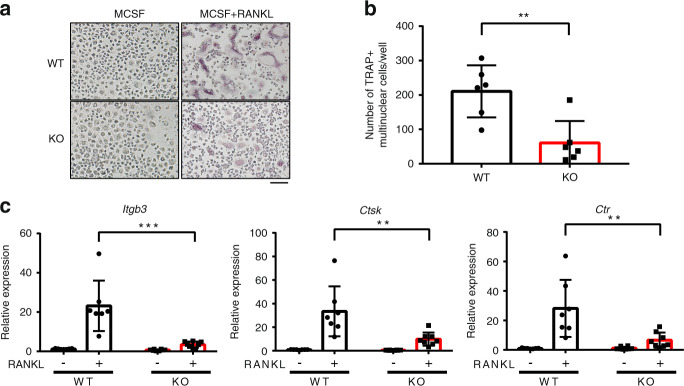
MEF2C-deficient cells show impaired osteoclastogenesis. **a** Representative image of TRAP staining of mouse osteoclasts. Scale bar, 100 μm. **b** Cumulative data showing numbers of osteoclasts from 6 independent experiments. **c** RT-qPCR analysis of *Itgb3*, *Ctsk* and *Ctr* mRNA after 72 h of culture with or without RANKL (50 ng·mL^−1^) normalized relative to *Hprt* mRNA. Control samples without RANKL were set at 1.0. WT; *n* = 7, KO; *n* = 8. Data are shown as mean ± SD. Statistics used: **b** Welch’s *t*-test, **c** Two-way ANOVA. **P < 0.01, ***P < 0.001

To corroborate our findings showing that osteoclast formation was suppressed in MEF2C^ΔMX^ mice, we harvested osteoclast precursor cells from MEF2C^ΔMX^ mice and differentiated them into osteoclasts in vitro as described previously.^28^ Effective deletion of MEF2C was observed in MEF2C^ΔMX^ OCPs and bone marrow-derived cells by immunoblot and immunofluorescence analysis (Supplementary Fig. 2e, f). In agreement with the data from in vivo bone mass and human OCPs, MEF2C^ΔMX^ cells showed significantly decreased osteoclastogenesis compared to control cells (Fig. 3a, b). The expression of osteoclast marker genes was also significantly lower in MEF2C^ΔMX^ cells compared to control cells (Fig. 3c). Taken together, our results support a positive role for MEF2C in both human and mouse osteoclastogenesis.

### Transcriptomic analysis identifies genes regulated by MEF2C

**Fig. 4:**
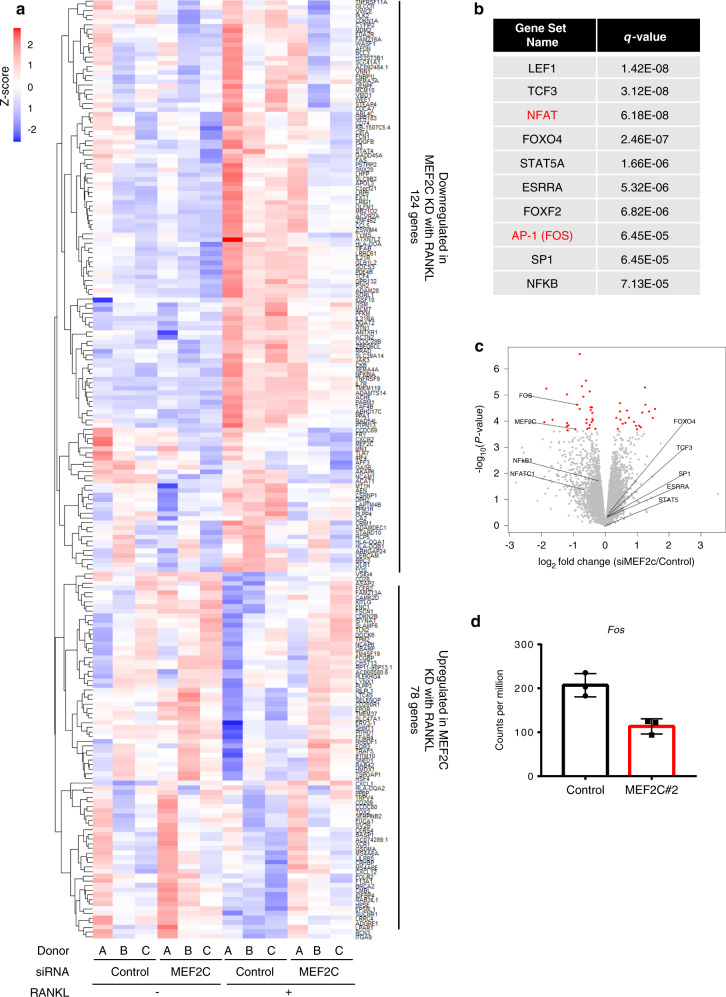
MEF2C regulates expression of *FOS* and c-FOS-target genes in RANKL-stimulated human OCPs. **a** Heatmap showing relative expression (z-score) of 202 genes differentially expressed with *P* < 0.01 in RANKL-stimulated MEF2C KD cells versus RANKL-stimulated control cells from three biological replicates. **b** Enriched transcription factor binding motifs in region ±2 kb relative to transcription start site in RANKL-regulated MEF2C-dependent genes, by gene set enrichment analysis (GSEA). **c** Volcano plot of RNA-seq analysis of differentially expressed genes in human macrophages transduced with control or MEF2C#2 siRNAs. MEF2C and eight other genes obtained from **b** are marked. Of note, LEF1 and FOXF2 were filtered out due to low expression level (CPM < 3). Red dots, genes with *q* value < 0.05 (34 genes). **d** Cumulative values for *FOS* in RANKL-stimulated samples from RNA-sequencing with three biological replicates. Data are shown as mean ± SD

“To gain insight into the mechanisms by which MEF2C suppresses human osteoclastogenesis, we performed an unbiased transcriptomic analysis using RNA-seq to identify genes whose expression was affected by MEF2C knockdown”.^28^ The expression of 202 genes was significantly altered by MEF2C-specific siRNA compared to control siRNA in human OCPs at 24 h after RANKL stimulation; 78 genes were upregulated, and 124 genes were downregulated in MEF2C KD early osteoclasts (Fig. 4a and Supplementary Table S1). 86 of 124 downregulated genes in MEF2C KD cells were RANKL-inducible genes, suggesting that MEF2C is involved in the early stage of RANKL-mediated responses. We found that MEF2C KD also regulated genes in OCPs (Fig. 4a, columns 1-3 versus columns 4-6). Pathway analysis of DEGs in OCPs indicated that MEF2C regulated genes related to inflammatory pathways, but not genes in pathways related to cell death or osteoclast differentiation (Supplementary Fig. S3d). We also tested whether MEF2C regulates protein expression of RANK and proximal RANKL-induced signaling pathways, which activates MAPK and NFκB signaling pathways.^29^ MEF2C-deficiency did not notably affect the protein expression of RANK and proximal RANKL-induced signaling pathways (Supplementary Fig. S3a–c). To uncover the mechanisms behind the involvement of MEF2C in osteoclastogenesis, we investigated the transcription factor binding motifs enriched in MEF2C regulated genes in early osteoclasts, using gene set enrichment analysis (GSEA). As shown in Fig. 4b, the top ten transcription factors included key transcription factors for osteoclast differentiation,^1^ such as NFATc1 and AP-1. Among these transcription factors, only mRNA amounts of c-Fos, which comprise AP-1 complexes with Jun family members, were significantly decreased by the gene-silencing of MEF2C (FDR < 0.05) (Fig. 4c, d). These results suggest c-Fos is one of the targets of MEF2C during osteoclastogenesis.

### MEF2C controls c-Fos expression

**Fig. 5:**
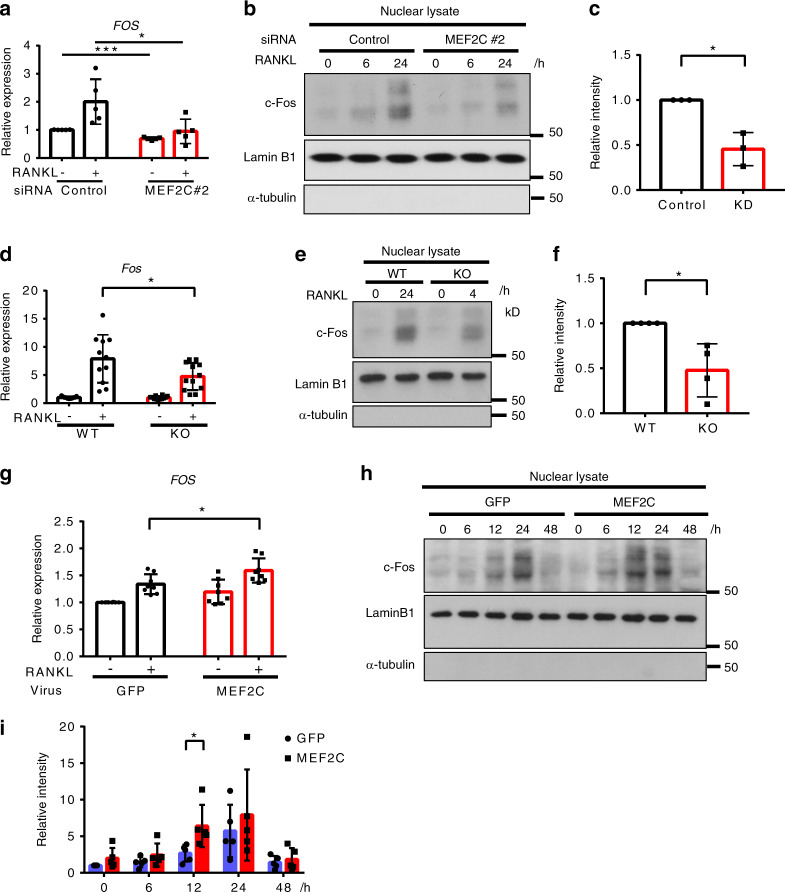
c-FOS mRNA and protein expression are dependent on MEF2C in human and mouse OCPs. Human osteoclast precursor cells were nucleofected with control or MEF2C #2 siRNAs. **a** RT-qPCR analysis of human *FOS* mRNA after 24 h of culture with or without RANKL normalized relative to *TBP* mRNA (control samples without RANKL set at 1.0). *n* = 5. **b** Representative immunoblotting of c-FOS in nuclear lysates. Lamin B1 and α-tubulin were used as controls for nuclear and cytoplasmic proteins, respectively. **c** Densitometric quantitation of c-FOS band intensity after 24 h of culture with RANKL from three independent donors. Mouse osteoclast precursor cells from MEF2C^ΔMX^ KO or littermate control WT mice were cultured with M-CSF and RANKL. **d** RT-qPCR analysis of mouse *Fos* mRNA after 24 h of culture with or without RANKL (50 ng·mL^−1^) normalized relative to *Hprt* mRNA (control samples without RANKL set at 1.0). WT; *n* = 11, KO; *n* = 12. **e** Representative images of immunoblotting analysis of c-FOS expression in nuclear lysates. Right panel, densitometric quantitation of band intensity from four samples of each genotype. **f** Densitometric quantitation of c-FOS band intensity (*n* = 4). Human osteoclast precursor cells were transduced with adenoviral particles encoding GFP or MEF2C-FLAG and stimulated with RANKL. **g** RT-qPCR analysis of human *Fos* mRNA after 12 h of culture with or without RANKL (40 ng·mL^−1^) normalized relative to *TBP* mRNA (control samples without RANKL set at 1.0). *n* = 8. **h** Immunoblotting of c-FOS at the indicated times. Representative images from five independent experiments. **i** Densitometric quantitation of c-FOS band intensity from five independent experiments. Data are shown as mean ± SD. Statistics used: **a, d, g, i** repeated measurement two-way ANOVA, **c, f** paired *t*-test. *P < 0.05, **P < 0.01

We next measured c-Fos mRNA and protein amounts in MEF2C KD cells. c-Fos has been shown to be indispensable for osteoclast formation and as expected c-Fos mRNA and nuclear protein amounts were induced in the early phase (within 24 h) of RANKL stimulation (Fig. 5a–c and Supplementary Fig. S4a).^19–21^ The expression level of c-Fos mRNA was significantly decreased in MEF2C KD cells both in the presence and absence of RANKL stimulation, and nuclear c-Fos expression decreased at 24 h after RANKL stimulation in MEF2C KD cells relative to control cells (Fig. 5a–c). Decreased c-Fos mRNA was observed in MEF2C^ΔMX^ cells compared to control cells (Fig. 5d). A significant role of MEF2C in augmenting nuclear c-FOS expression was corroborated using MEF2C^ΔMX^ cells compared to control cells (Fig. 5e, f). To further support the regulation of RANKL-induced c-FOS by MEF2C, we used a gain-of-function approach to ectopically express MEF2C (or control GFP) in human OCPs using adenoviral-mediated transduction. Consistent with our observations in MEF2C KD cells, increased MEF2C expression enhanced both c-FOS nuclear protein and mRNA expression, which peaked at 12 h after RANKL stimulation (Fig. 5g–i). Collectively the results indicate that MEF2C positively regulates c-Fos expression.

**Fig. 6:**
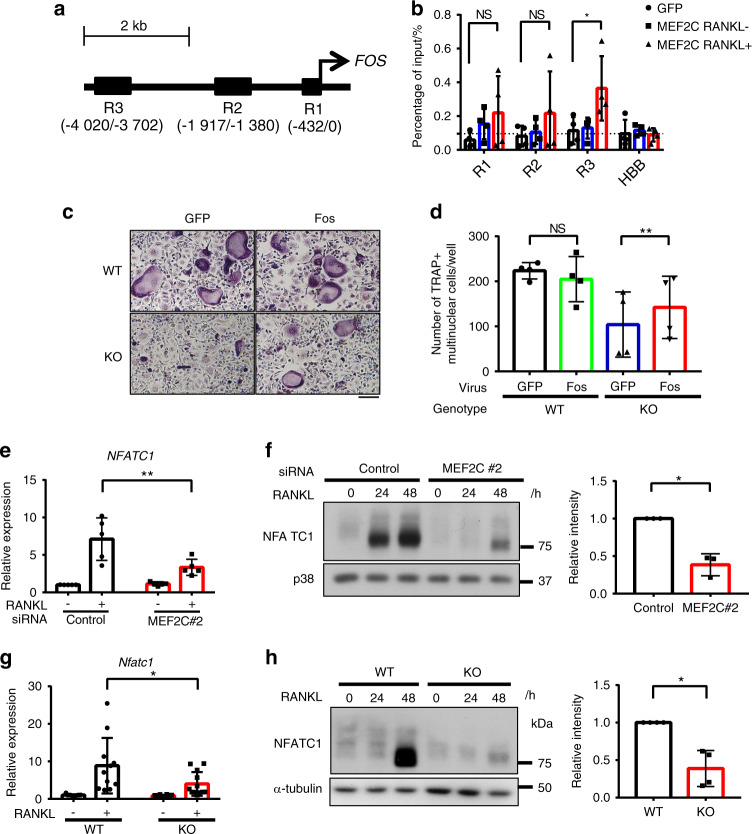
MEF2C binds to the upstream region of *FOS* gene and regulates NFATC1 expression. **a** A schematic view of *FOS* upstream regions with putative MEF2C binding sites predicted based on the analysis of MEF2C ChIP-sequencing data. **b** ChIP-qPCR analysis of the three putative MEF2C binding sites in the *FOS* upstream region. MEF2C binding was assessed in human OCPs transduced with adenoviral particles encoding GFP or MEF2C-FLAG and immunoprecipitated with FLAG antibodies. Dotted line represents enrichment level in the negative control *HBB* region in GFP transduced samples. (*n* = 4) Mouse osteoclast precursor cells from MEF2C^ΔMX^ KO or littermate control WT mice were transduced with retroviral particles encoding GFP or FOS and cultured with M-CSF and RANKL. **c** Representative image of TRAP staining of mouse osteoclasts. Scale bar, 100 μm. **d** Cumulative data showing numbers of osteoclasts from four independent experiments. Human osteoclast precursor cells were nucleofected with control or MEF2C #2 siRNAs. **e** RT-qPCR analysis of human *NFATC1* mRNA after 48 h of culture with or without RANKL normalized relative to *TBP* mRNA (control samples with RANKL set at 1.0). *n* = 5. **f** Representative immunoblot of human NFATc1. p38 was used as a loading control. Right panel, densitometric quantitation of band intensity from three donors. Mouse osteoclast precursor cells from MEF2C^ΔMX^ mice or littermate control WT mice were cultured with M-CSF and RANKL. **g** RT-qPCR analysis of mouse *Nfatc1* mRNA after 24 h of culture with or without RANKL (50 ng·mL^−1^) normalized relative to *Hprt* mRNA (control samples without RANKL set at 1.0). WT; *n* = 11, KO; *n* = 12. **h** Representative images of immunoblotting analysis of mouse NFATc1 expression. Right panel, densitometric quantitation of band intensity from 4 samples of each genotype. Data are shown as mean ± SD. Statistics used: **e** repeated measurement two-way ANOVA, **f** paired *t*-test, **g** two-way ANOVA, **h** Welch’s *t*-test. *P < 0.05, **P < 0.01, ***P < 0.001

As the effect of MEF2C-deficiency on proximal RANKL-induced signaling pathways was minimum (Supplementary Fig. S3b, c), an alternative mechanism of regulation is that MEF2C directly binds to regulatory regions of *FOS* to promote transcription. To test this hypothesis, we used ChIP-qPCR to assess MEF2C binding to regulatory regions of *FOS*. Limitations related to the affinity of antibodies against endogenous MEF2C and numbers of human primary OCPs that could be obtained did not allow for reliable immunoprecipitation. As an alternative approach, we utilized human OCPs adenovirally-transduced to express FLAG-tagged MEF2C, where high affinity and specificity FLAG antibodies enable detection of strong and specific signals in ChIP-qPCR assays. We identified three potential MEF2C binding sites, termed R1–R3, in the upstream region of the c-FOS promoter based on the publicly available ENCODE MEF2C ChIP-seq database obtained using the GM12878 cell line, an established lymphoblastoid cell line^30^ and we also confirmed that the potential MEF2C binding sites were regions of open chromatin (detected by ATAC-seq) and were bound by PU.1, a lineage-determining transcription factor in human OCPs^31^ (Fig. 6a, Supplementary Fig. S5a), suggesting that these sites correspond to regulatory regions.

In the absence of RANKL stimulation there was minimal occupancy of FLAG-MEF2C relative to the negative control beta hemoglobin (HBB) promoter^32^ at c-FOS upstream regions in cells transduced to express FLAG-MEF2C versus GFP controls (Fig. 6b). Among the potential binding sites, FLAG-MEF2C occupancy significantly increased at the R3 region after RANKL stimulation (Fig. 6b). These results suggest that MEF2C binds to the upstream region of *FOS* in response to RANKL.

To test the functional importance of MEF2C-mediated regulation of c-FOS, we performed a complementation assay by forcibly expressing c-FOS in MEF2C-deficient OCPs. MEF2C-deficient OCPs were transduced with retroviral particles encoding c-FOS or negative control GFP (Supplementary Fig. S5b). Forced expression of c-FOS significantly increased generation of TRAP^+^ multinucleated cells from MEF2C-deficient OCPs but did not fully restore the differentiation defect relative to control OCPs (Fig. 6c, d); c-FOS also induced formation of bigger osteoclasts in MEF2C-deficient OCPs (Fig. 6c). However, c-FOS only modestly, albeit significantly, increased expression of NFATC1, and did not significantly increase expression of other osteoclast marker genes in MEF2C-deficient cells (Supplementary Fig. S5c–f). These results support that c-FOS mediates part of the effects of MEF2C on osteoclast differentiation, but suggest additional MEF2C target genes are required for implementation of the full osteoclastogenesis program.

### NFATC1 induction is dependent on MEF2C

NFATc1 is a master regulator required for osteoclastogenesis whose expression is induced by RANKL-activated upstream factors such as c-FOS.^19,21^ Consistent with prior observations, NFATc1 was induced by RANKL in a time-dependent manner (Supplementary Fig. S6a). Strikingly, knockdown of MEF2C in human OCPs or MEF2C deficiency in mouse OCPs strongly attenuated RANKL-induced NFATc1 mRNA and protein expression (Fig. 6e–h). In contrast, forced MEF2C expression resulted in increasing expression of NFATc1 mRNA and protein (Supplementary Fig. S6b–d). These results link the MEF2C-c-FOS axis with induction of NFATC1 expression, thereby providing an explanation for diminished osteoclastogenesis when MEF2C expression is abrogated.

### MEF2C deficiency protects mice from pathological bone erosion in inflammatory arthritis

**Fig. 7:**
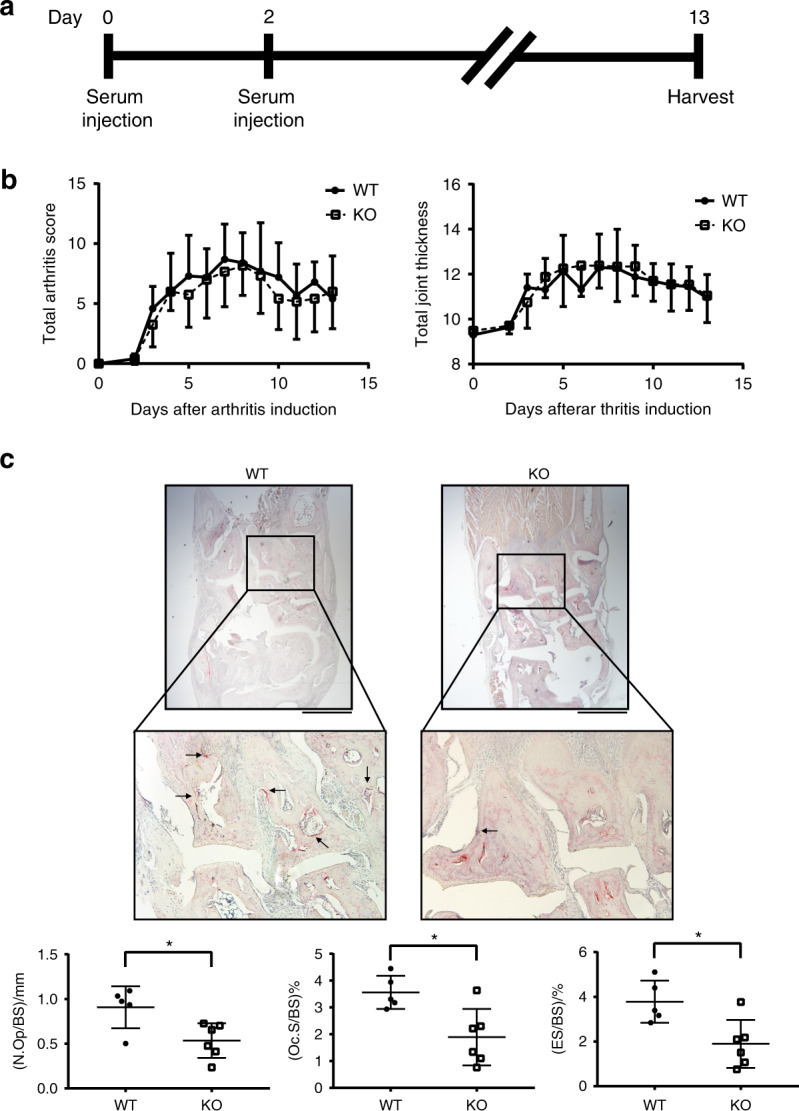
MEF2C deficient mice show attenuated pathological bone resorption in the K/BxN serum transfer arthritis model. **a** Schematic of experiments. **b** Time course of joint swelling and clinical score of K/BxN serum transfer arthritis in WT and MEF2C^ΔMX^ KO mice. WT; *n* = 5, KO; *n* = 6. **c** TRAP staining of histological sections of hind paw and histomorphometric analysis of tarsal bones. Arrow, osteoclasts. Scale bars, 400 μm. Data are shown as mean ± SD. Statistics used: **c** Welch’s *t*-test *P < 0.05

Given the positive role of MEF2C in osteoclastogenesis, we used publicly available dataset (GEO: GSE97779)^33^ and examined the expression of MEF2C in synovial CD14^+^ cells from patients with rheumatoid arthritis, which exhibits elevated pathological osteoclast-mediated bone erosion. Synovial CD14^+^ cells are able to differentiate into osteoclasts.^34^ Joint osteoclast precursor cells from RA exhibited increased expression of MEF2C (Supplementary Fig. S7). To address the importance of MEF2C in osteoclast-mediated pathological bone resorption, we tested the effects of MEF2C deficiency on bone loss in K/BXN serum-induced arthritis.^35^ K/BxN serum was administrated intra-peritoneally on day 0 and day 2 (Fig. 7a). The severity of arthritis, as assessed by clinical score and ankle joint thickness until day 13, was comparable between littermate control and MEF2C^ΔMX^ mice (Fig. 7b). However, histomorphometric analysis revealed that osteoclast number, osteoclast surface area, and eroded surface area were significantly decreased in MEF2C^ΔMX^ mice compared to control mice (Fig. 7c), indicating that under similar levels of inflammation, MEF2C-deficiency clearly affected osteoclast-mediated bone erosion. Our results suggest a role for MEF2C in osteoclast activation and pathological bone loss in inflammatory conditions.

## Discussion

A role for MEF2C in the regulation of bone development has been proposed on the basis of defects in MEF2C heterozygous mice,^11^ GWAS in patients with osteoporosis,^6–8^ and its role in regulating SOST expression.^15,16,36^ However, despite extensive study of the function of MEF2C in bone formation and mineralization, the importance of MEF2C in regulating osteoclasts and related bone resorption remains unclear. Here, we provide evidence that MEF2C acts as a positive regulator of osteoclastogenesis and promotes osteoclast formation and function in vitro and in vivo. MEF2C deficiency in primary OCPs resulted in decreased RANKL-induced osteoclastogenesis, and increased bone mass in vivo with no alterations in osteoblast number and function. We also show that MEF2C contributed to arthritic bone erosion in an inflammatory arthritis model. Thus, our data demonstrate a new positive role of MEF2C in promoting physiological and pathological bone erosion.

MEF2C was initially identified as an important controller of muscle and neural development and is also involved in differentiation processes in many cell types.^37,38^ The phenotype of MEF2C heterozygous mice reveals that MEF2C plays an important role in bone development.^11^ In addition, SNPs in the MEF2C locus are strongly associated with bone mineral density.^6,7,39–41^ The role of MEF2C in osteocytes, osteoblasts, and chondrocytes, which affect bone development, has been investigated using mice with conditional deletion of MEF2C.^15^ Our results suggest that the role of MEF2C in osteoclasts may contribute to bone phenotypes that have been observed in patients with allelic variants in the *MEF2C* locus. Our in vivo approach employed an inducible MEF2C deletion system using MX1 cre that deletes strongly in hematopoietic bone marrow cells and avoids potential developmental defects that cause MEF2C-germline deletion mice to die at embryonic day 9.5. Although we did not observe an osteoblast defect in these experiments, it is possible that deletion of MEF2C in other cell types contributed to the in vivo phenotypes. Strikingly, deleting MEF2C for a relatively short time (~10 weeks) significantly decreased in vivo osteoclast number and activity, resulting in increased bone mass. Moreover, arthritic bone erosion also significantly decreased by MEF2C deficiency. These results suggest that the *MEF2C* locus influences bone development and osteoporosis by coordinating the function of many different cell types in bone, and such fine-tuning of bone remodeling by MEF2C provides new insights into bone metabolism.

Our findings introduce MEF2C as a new, positive regulator in the transcriptional factor network upstream of NFATc1 in osteoclastogenesis. MEF2C is a transcription factor that can bind to DNA directly or indirectly as part of protein complexes that include DNA-binding partners such as c-FOS. The transcriptional activity and chromatin-binding of MEF2C are regulated by calcium-mediated signaling pathways^23^ and MEF2C can function as a transcriptional activator or inhibitor depending upon context and its interaction partners. The potential mechanism of the inhibitory action of MEF2C is for MEF2C to interact with HDACs to generate a repressed state on chromatin and suppress gene expression. The HDAC4-MEF2C axis has been established in cancer and cartilage,^11,42^ and a recent study also suggested a role for HDAC5 in attenuating MEF2C transcriptional activity at the sclerostin gene in osteocytes.^43^ Thus, although, in accord with a positive transcriptional function, deletion of MEF2C in osteoblasts/osteocytes results in diminished SOST expression,^15^ interaction of DNA-bound MEF2C with HDACs can restrain the positive effects of MEF2C on transcription. Interestingly, stimulation with PTH can release HDACs and thereby promote MEF2C transcriptional activity. Our transcriptomic studies showed that MEF2C has both positive and negative transcriptional functions in OCPs that have been stimulated with RANKL. While MEF2C expression was diminished by RANKL stimulation, MEF2C expression persisted during osteoclastogenesis. RANKL, which induces calcium signaling that has been shown to regulate MEF2C in other cell types, induced translocation of MEF2C to the promoter of c-FOS. Overexpression of MEF2C is not sufficient for c-FOS expression, suggesting that RANKL signals are required for the MEF2C-mediated c-FOS induction. How RANK signaling regulates MEF2C function, and the potential role of HDACs in MEF2C-mediated gene repression will be the subject of future investigation. Our data also suggest that MEF2C-induced genes in addition to c-FOS are important for osteoclastogenesis, and that regulation of NFATc1 expression by MEF2C may be indirect and mediated by c-FOS in cooperation with additional MEF2C-induced transcription factors.

A previous report shows that MEF2C is capable of forming a complex with c-FOS to regulate MMP13 expression in osteoblastic cells. Therefore, it is possible that, in addition to direct binding of MEF2C to its cognate target motif, MEF2C may regulate downstream targets by binding to AP-1 motifs as a complex with c-FOS. Coordinate binding of MEF2C and c-FOS at the *Nfatc1* locus may explain the nearly complete loss of expression of NFATc1, a major target of c-FOS, in MEF2C KD cells. However, we are unable to identify the binding of MEF2C in the upstream regions of *NFATc1* locus (data not shown), suggesting that MEF2C may indirectly control the regulatory regions of NFATc1 by forming a complex with c-FOS or by activating other signaling pathways. Consistent with this idea, ectopic c-FOS expression restored impaired osteoclastogenesis in terms of number of osteoclasts generated. However, the size of osteoclasts was small in MEF2C-deficient cells in which c-FOS expression was restored. These results suggest that MEF2C activates not only the c-FOS/NFATc1 axis but also other pathways to regulate the fusion of OCPs, which requires further investigation.

MEF2C is highly expressed in OCPs, and its expression decreases but persists after RANKL stimulation. During osteoclastogenesis, RANKL-induced c-FOS activates IFNβ and IFNβ-induced genes later suppress c-FOS expression.^21^ Since MEF2C expression is suppressed by IFNs (unpublished observations, Fuji et al.),^21^ and induces c-FOS, transient expression of MEF2C could be part of IFNβ-mediated negative feedback regulation of osteoclastogenesis. It is also possible that other MEF2 family members may replace the function of MEF2C at the later stages of osteoclastogenesis. For example, it has been shown that MEF2A positively regulates the Atp6v0d2 gene after RANKL stimulation in the mouse macrophage RAW264.7 cell line.^44,45^

In the early stage of postmenopausal osteoporosis, a decrease in estrogen levels results in increased osteoclastogenesis, which perturbs bone remodeling by accelerating bone loss and subsequently leads to a rapid decrease in bone mineral density.^4,46,47^ Given that MEF2C positively regulates osteoclastogenesis, SNPs in the MEF2C locus can be associated with changes in osteoclast activity that contribute to the pathogenesis of postmenopausal osteoporosis, which requires further investigation. In summary, this study delineates mechanisms by which MEF2C regulates osteoclastogenesis, revealing MEF2C as a positive regulator of osteoclastogenesis that contributes to physiological and pathological bone remodeling.

## Materials and methods

### Reagents

Human M-CSF, sRANKL, and TNF were purchased from Peprotech (Rocky Hill, NJ, USA). The antibodies used for immunoblotting are as follows: NFATc1 (#sc-7294), RANK (#sc-37436) (Santa Cruz Biotechnology, Dallas, TX, USA); MEF2C (#5030), c-Fos (#2250), IkBα (#9242), p-p38 (#9215), p-ERK1/2 (#9101), ERK1/2 (#9102), p65 (#4764), p105/p50 (#3035), and p38 (#9212) (Cell Signaling Technology, Danvers, MA, USA); Lamin B1 (Abcam #16048, Cambridge, UK); α-tubulin (Sigma-Aldrich #T9026, St. Louis, MO, USA); FLAG (for immunoblotting: Biolegend #637301, San Diego, CA, USA. For ChIP: Sigma-Aldrich (#1804); HRP-conjugated anti-mouse IgG (Jackson ImmunoResearch Laboratories #115-035-003, West Grove, PA, USA); HRP-conjugated anti-rabbit IgG (GE Healthcare #NA9310V, Chicago, IL, USA); HRP-conjugated anti-rat IgG (Jackson ImmunoResearch Laboratories #112-035-003). The antibodies used for flow cytometry are as follows: CD34-Biotin (Biolegend #128604), c-kit-Biotin (Biolegend #105804), Ter119 (Biolegend #116204), CD3-APC/Cy7 (Biolegend #100330), B220-PE (BD Biosciences #553090, San Jose, CA, USA), CD45-APC R700 (BD Biosciences #565478), CD11b-BV421 (Biolegend #101235), Ly6G-BV650 (Biolegend #127641), Ly6C-BV510 (Biolegend #128033), F4/80-PE/Cy7 (Biolegend #123114), Streptavidin-PerCP/Cy5.5 (Biolegend #405214) and DAPI (Thermo Fisher Scientific #D1306, Waltham, MA, USA). For ChIP experiments, anti-FLAG M2 antibody (Sigma-Aldrich #F1804) was used. Anti-MEF2C antibody (Aviva Systems Biology #OAGA02537, San Diego, USA), Rabbit IgG (Control Antibody) (Vector Laboratories #I-1000, Burlingame, CA,USA), Citrate Buffer pH 6.0 (Sigma-Aldrich #C9999-1000ML), VECTASTAIN Elite ABC HRP Kit (Peroxidase, Rabbit IgG) (Vector Laboratories), and DAB (Vector Laboratories #SK-4100) were used for immunohistochemistry. For immunofluorescence, anti-MEF2C antibody (Cell Signaling Technology #5030), Alexa Fluor 488 anti-rabbit IgG (Thermo Fisher Scientific #A11034), Biotinylated goat anti-rabbit IgG antibody (Vector Laboratories #BA-1000), Alexa Fluor 594 Streptavidin (Biolegend #405240), and Fluoroshield Mouting Medium (Abcam #ab104139) were used.

### Human osteoclast differentiation

“Peripheral blood mononuclear cells were obtained from blood leukocyte preparations purchased from the New York Blood Center, by density gradient centrifugation with Lymphoprep (Stemcell Technology, Vancouver, BC, Canada) using a protocol approved by the Hospital for Special Surgery institutional review board”.^48^ “CD14^+^ monocytes were obtained from peripheral blood, using antihuman CD14 magnetic beads, as per the manufacturer’s protocol (Miltenyi Biotec, Auburn, CA, USA). Purity of monocytes was >97%, as verified by flow cytometric analysis. CD14 ^+^ cells were plated at a density of 1 × 10^6^ cells·mL^−1^ and cultured with 20 ng·mL^−1^ of M-CSF (Peprotech) in alpha modified essential medium (α-MEM) (Thermo Fisher Scientific) supplemented with 10% Hyclone fetal bovine serum (GE Healthcare) and 1% L-glutamine (200 mmol·L^−1^, Thermo Fisher Scientific) for 1 day to obtain OCPs”.^25^ OCPs then were incubated with 20 ng·mL^−1^ of M-CSF and 40 ng·mL^−1^ of human soluble RANKL (Peprotech) to differentiate into osteoclasts. “Medium and cytokines were replenished every 3 days. When multinucleated cells were observed, cells were fixed and stained for TRAP using the Acid Phosphatase Leukocyte diagnostic kit (Sigma Aldrich) as recommended by the manufacturer. Multinucleated (>3 nuclei), TRAP-positive osteoclasts were counted in triplicate wells”.^48^

### RNA interference

For short interfering RNA (siRNA) experiments, 10^7^ human CD14^+^ cells were nucleofected with 0.32 nmol of siRNA oligonucleotides using a Nucleofector kit (Lonza, Basel, Switzerland) as previously described.^48^ “Human Monocyte Nucleofector buffer (Lonza) and the AMAXA Nucleofector System program Y001 for human monocytes were used according to the manufacturer’s instructions. We tested two different sets of siRNAs and MEF2C-specific (#4392420 ID8652 for MEF2C #1 and ID 8653 for MEF2C #2) and control (#4390843) siRNAs were obtained from Thomas Fisher Scientific”.^25^

### Virus transduction

“For adenoviral transduction, recombinant adenoviral particles encoding human MEF2C-FLAG and control adenoviral particles encoding green fluorescent protein (Ad-CMV-GFP) were purchased from Vector Biolabs (Malvern, PA, USA). Human CD14^+^ cells were incubated at a density of 1.5 × 10^6^ cells per mL for 6 days with M-CSF (40 ng·mL^−1^) on six well plates in α-MEM medium supplemented with 10% of fetal bovine serum (FBS) (GE Healthcare) and 1% L-glutamine (200 mmol·L^−1^, Thermo Fisher Scientific). Cells were washed and incubated in low-serum media (2% FBS) with 20 ng·mL^−1^ of MCSF and adenoviral particles (100 particles per cell) overnight, and then used for experiments”.^25^

For retroviral transduction, the retroviral vectors encoding c-Fos (pMX-Fos-eGFP) or GFP (pMX-eGFP) were kindly provided by Dr. Hong-Hee Kim (Seoul National University, Korea). “The vectors were transfected into packaging cell line Plat-E using FuGENE HD Transfection Reagent (Promega, Madison, WI, USA), and then the viral supernatant was collected after 48 h of incubation. The filtered virus-containing supernatant was added to the OCPs in the presence of 6 μg·mL^−1^ polybrene (Santa Cruz Biotechnology) and cells were incubated for an additional 48 h. Cells were then used for experiments”.^28^

### Mouse models

All animal procedures were approved by the Weill Cornell Medical College IACUC. Mice with inducible deletion of *Mef2c* were obtained by crossing MEF2C^*flox/flox*^ mice (The Jackson Laboratory, Bar Harbor, ME, USA) with mice with a Mx1-promoter-driven Cre transgene on the C57/BL6 background (known as Mx1-cre; The Jackson Laboratory). Mef2c^*flox/flox*^ Mx1cre^+^ mice (referred to as MEF2C^ΔMX^ mice) and littermate control Mef2c^*WT/WT*^ Mx1cre^+^ mice were used for the experiments. To induce *Mef2c* deletion, 300 μg of Poly (I:C) (Thermo Fisher Scientific) was injected three times at age of 6 weeks. Poly (I:C) induces type I IFNs that activate the promoter of Mx1 (Myxovirus Resistance Protein 1), interferon inducible dyamin-like GTPase, which drives the Cre transgene, enabling cre recombinase expression to delete MEF2C. The bone phenotype of 16-week-old male WT and MEF2C KO mice was assessed using a Scanco micro-CT-35 instrument (Scanco Medical, Bruttisellen, Switzerland) as previously described.^49^ “The femurs were fixed in 4% paraformaldehyde overnight, decalcified with 10% neutral buffered EDTA (Sigma-Aldrich), and then embedded in paraffin. To assess in vivo osteoclastogenesis, sections were stained with TRAP (tartrate-resistant acid phosphatase) and methyl green for osteoclast visualization. All measurements were performed using OsteoMeasure software (OsteoMetrics. INC) using standard procedures”.^25,50^ Briefly, the number of osteoclasts was calculated as the number of TRAP^+^ cells that were multinucleated (>3 nuclei) and adjacent to bone. Bone surface (BS) was defined as the length of the bone surface of the secondary sponginosa. The number of osteoclasts (N.OC/BS) was evaluated as the TRAP^+^ multinucleated cells adjacent to bone normalized by the length of the bone surface. Osteoclast surface/bone surface (Oc.S/BS) was calculated as the length of the TRAP^+^ osteoclast surface facing bone marrow, measured and subdivided by BS. Eroded surface/bone surface (ES/BS) was calculated as the length of the TRAP^+^ osteoclast surface adjacent to bone, measured and subdivided by the length of BS. Measurements were restricted to the secondary spongiosa between 100 and 2 000 μm distal to the growth plate metaphyseal junction of the distal femur.

### K/BxN serum transfer arthritis model

For arthritis experiments, K/BxN serum pools were prepared as described previously.^51^ All animals were randomly assigned into experimental groups. Arthritis was induced in 10-week-old male mice by intraperitoneal injection of K/BxN serum (100 μL on day 0 and 80 μL on day 2). “The development of arthritis was monitored by measuring the thickness of wrist and ankle joints using a digital caliper and scoring wrist and ankle joints. For each animal, joint thickness was calculated as the sum of the measurements of both wrists and both ankles”.^25^ “The severity of arthritis was scored in a blinded fashion by two investigators for each paw on a 3-point scale, in which 0 = normal appearance, 1 = localized edema/erythema over one surface of the paw, 2 = edema/erythema involving more than one surface of the paw, and 3 = marked edema/erythema involving the whole paw.^25^ The scores of all four paws were added for a composite score”.^25^ “For histopathologic assessment, mice were euthanized and hind paws were harvested and fixed in 4% paraformaldehyde overnight. These samples were decalcified with 10% neutral buffered EDTA (Sigma-Aldrich) and embedded in paraffin”.^48^ Two sections from different areas (at least >50 μm of distance from each other) were stained with TRAP and hematoxylin for osteoclast visualization. Histomorphometric analysis was performed with the same method as the femur, but counting osteoclasts which are located at the surface of bones from at least seven tarsal joints. All parameters were taken on an average of two slides.

### Dynamic bone labeling

“To measure bone mineralization, mice were intraperitoneally injected with Calcein (green;Sigma) at 5 μg·g^−1^ (body weight) twice, at 7 days apart. Two days after the second injection, femurs were collected, fixed in 4% paraformaldehyde, and embedded in OCT compound (Thermo Fisher Scientific, Waltham, MA) as described previously”,^28^ and then cut into 7-μm frozen sections that were prepared using the Kawamoto tape method as described in.^52^ Histomorphometry analysis using OsteoMetrics software (OsteoMeasure) was performed on trabecular bone within the femoral metaphysis. “Mineral apposition rate (MAR) was determined by measuring the distance between two fluorochrome-labeled mineralization fronts. The mineralizing surface was determined by measuring the double-labeled surface and half of the single-labeled surface, expressing this value as a percentage of total bone surface. The bone formation rate was expressed as MAR × mineralizing surface/total bone surface, using a surface referent”.^28^

### Mouse osteoclast differentiation

“Bone marrow cells were flushed out from the femurs, followed by lysis of red blood cells using ammonium chloride potassium lysis buffer (Thermo Fisher Scientific). The surviving cells were cultured in α-MEM, supplemented with 10% FBS, 1% penicillin–streptomycin (Thermo Fisher Scientific) and 5% L929 cell supernatant, which served as a source of M-CSF.^28^ The nonadherent cell population was recovered the next day and cultured with M-CSF-containing conditioned medium (CM) for three additional days. We defined this cell population as mouse OCPs. Mouse OCPs were plated at a seeding density of 1.5 × 10^5^ per mL and incubated with M-CSF-containing CM and RANKL (50 ng·mL^−1^), with exchange of fresh M-CSF-containing CM and RANKL every 2 days. When multinucleated cells were observed, cells were fixed and stained with the TRAP staining kit”.^28^

### Bone resorption pit assay

Cells were seeded in Osteo Assay Surface Plate (Corning, Corning, NY) and cultured in the presence of M-CSF and RANKL. After confirming osteoclast formation and pit formation around cells under a microscope, typically 3–5 days of culture with RANKL, cells were removed twice, with 10% bleach solution for 5 min at room temperature, followed by washing with distilled water. Plates were stained with 1% toluidine blue solution for 10 s to visualize the formation of pits. Total area of pits was analyzed through ImageJ v1.52a with Fiji plugin package.

### RNA and quantitative Real-Time PCR (RT-qPCR)

“Total RNA was extracted from cells using RNeasy Mini kit (QIAGEN, Venlo, Netherlands) and 300 ng of total RNA was reverse transcribed using the RevertAid First Strand cDNA Synthesis kit (Thermo Fisher Scientific). RT-PCR was performed in duplicate with Fast SYBR Green Master Mix and QuantoStudio 5 Real-time PCR system (Applied Biosystems, Foster City, CA, USA)”.^25^ Primer sequences are provided in the Table S2. Transcript levels were calculated by the 2^−*ΔΔCT*^ method^53^ and normalized relative to corresponding housekeeping genes (mouse *Hprt* or human *TBP*) in each sample.

### Immunoblotting analysis

For immunoblotting, “whole cell lysates or nuclear lysates were fractionated on 7.5% polyacrylamide gels using SDS-PAGE and transferred to polyvinylidene difluoride membranes for probing with antibodies”.^25^ Densitometric quantitation was performed with ImageJ v1.52a.

### Cell viability assay

Human OCPs were seeded in 96 well plates at the density of 1.5 × 10^5^ cells per mL after adenovial transfection. Cells were incubated with MCSF (20 ng·mL^−1^) and RANKL (40 ng·mL^−1^) for indicated times, and then incubated with XTT Cell Proliferation Assay Kit (ATCC 30-1011K, Manassas, VA, USA) for 3 h following the manufacturer’s instructions.

### Flow cytometry

The femur and tibia were digested with Collagenase A, Dispase II, and DNAse for 15 min and then cells were filtered with cell strainer. Erythrocytes were lysed with ACK lysis buffer (Sigma-Aldrich). To block unspecific staining, cells were incubated with anti-mouse CD16/CD32 antibodies for 10 min on ice. Cells were stained with 1st antibodies including anti-mouse CD34, c-Kit, Ter119, CD45, CD3, B220, Ly6C, Ly6G, CD115, F4/80 (1:100), and CD11b (1:200) for 15 min, and for CD34, c-Kit, and Ter119, samples were further stained with Streptavidin-PerCP/Cy5.5 (1:500) for 10 min. Cell sorting was performed with Influx (BD Biosciences). All antibodies were purchased from BioLegend (San Diego, CA).

### Immunofluorescence

For in vitro cultures, cells were seeded in BD Falcon CultureSlides (BD Biosciences) and cultured with M-CSF and RANKL. For sorted cells by flow cytometry, cells were immobilized on slide glasses with Cytospin. Cells were fixed with 4% formaldehyde for 10 min and then blocked with 5%FBS/0.3%TritonX-100/PBS for 60 min. Anti-MEF2C antibody (Cell Signaling Technology) was added to cells in 1:400 dilution with 1%BSA/0.3%TritonX-100/PBS and incubated overnight at 4 °C. After washing with PBS, 1:1 000 diluted Alexa Fluor 488 anti-rabbit IgG (Thermo Scientific) or 1:300 diluted Biotinylated anti-rabbit IgG (Vector Laboratories) were incubated in 1%BSA/0.3%TritonX-100/PBS for 60 min at room temperature. Cells incubated with biotinylated anti-rabbit IgG were stained with 1:500 diluted streptavidin Alexa Fluor 594 for 30 min at room temperature. Cells were mounted with Fluoroshield Mounting Medium and observed using NIKON SMZ25 with CMOS camera (Zyla 5.5, Andor). Fluorescence intensity was evaluated with ImageJ v1.52a.

### Immunohistochemistry

Paraffin embedded distal femur was 7-μm sectioned and deparaffinized. Specimens were placed at 65 °C in citrate buffer pH 6.0 overnight for antigen retrieval. Specimens were then blocked with 5% Goat IgG free FBS/0.3%TritonX-100/PBS for 60 min and incubated with 1:500 diluted anti-MEF2C antibody (Aviva Systems Biology) in 1% IgG free BSA/0.3%TritonX-100/PBS overnight at 4 °C. After washing with PBS, VECTASTAIN Elite ABC HRP Kit (Peroxidase, Rabbit IgG) (Vector Laboratories #PK6101) was used as per the manufacturer’s protocol. DAB (Vector Laboratories) was applied for 3 min for development, and then nuclei were counterstained by methyl green. The images were captured by Aperio CS2 (Leica Biosystems, Buffalo Grove, IL, USA) and the number of DAB positive cells from joint cartilage to 1 mm proximal from growth plate was counted using QuPath v 0.2.0.

### RNA-sequencing

Three biological replicates from three independent donors were used for RNA-sequencing. “Total RNA was extracted using RNeasy mini kit (Qiagen). True-seq RNA Library preparation kits (Illumina) were used to purify poly-A^+^ transcripts and generate libraries with multiplexed barcode adapters following the manufacturer’s instructions. All samples passed quality control analysis on a Bioanalyzer 2100 (Agilent). Paired-end reads were obtained on an Illumina HiSeq 2500 in the Weill Cornell Medical College Genomics Resources Core Facility or the Weill Cornell Epigenomics Core Facility”.^25^ “Read quality was assessed with FastQC v0.11.6 and adapters trimmed using Cutadapt v1.15. Reads were then mapped to the human genome (hg38) and reads in exons were counted against Gencode v27 with STAR Aligner v2.5.3a. Differential gene expression analysis was performed in R v3.5.1 using edgeR v3.20.9. Genes with low expression levels (<3 cpm in at least one group) were filtered from all downstream analyses. The Benjamini–Hochberg false discovery rate procedure was used to calculate *q*-value. Genes with *P* value > 0.01 and log_2_ (fold-change) < 0.5 were filtered out”.^54^ A heatmap was generated from the averaged cpm using Pheatmap v1.0.12 with Euclidean hierarchical clustering. Representative upstream transcriptional factors in Fig. 4b and representative pathways enriched in OCPs (Supplementary Fig. S3d) were selected based on GSEA of C3 gene sets and gene ontology biological process, respectively.^55^

### Chromatin immunoprecipitation (ChIP)

“Adenoviral-transfected human OCPs (4.5 × 10^6^ cells) were cultured with or without 40 ng·mL^−1^ of RANKL for 6 h and then fixed by adding formaldehyde directly to the medium to a final concentration of 1% for 5 min. Cells were harvested, washed, and lysed. Chromatin was sheared by sonication using a Bioruptor sonicator (Diagenode, Denville, NJ, USA). Sheared chromatin was precleared and then immunoprecipitated with 1 μg of anti-FLAG M2 antibody (Sigma-Aldrich #F1804). Immune complexes were subsequently collected and washed, and DNA crosslinking was reversed by heating at 65 °C overnight. After proteinase K digestion (Roche, Basel, Switzerland), DNA was extracted using the PCR purification kit (Qiagen) and RT-PCR was performed to detect the occupancy of target proteins”.^48^ Beta hemoglobin (HBB) was not expressed in osteoclast precursor cells and was used as a negative control as previously described.^32^ Signals obtained from the ChIP were divided by signals obtained from an input sample. This input sample represents the amount of chromatin used in the ChIP. The primer sequences are listed in Supplementary Table S2.

### Statistical analysis

Power analysis was computed for the primary outcome of osteoclast number by setting the probability of a Type I error at 0.05, effect size at 4, and power at 0.80. All statistical analyses were performed with Prism 7.0 software (GraphPad Software, La Jolla, CA, USA) or R (ver. 3.6.0) using the two-tailed, paired *t*-test, unpaired Welch’s *t*-test, Wilcoxon signed-rank test (two conditions), one-way or two-way ANOVA, and two-way repeated measures ANOVA (RMANOVA) for multiple comparisons (more than two conditions) with post hoc Tukey’s correction. Shapiro–Wilk normality tests were performed, and for data that fell within Gaussian distribution, we performed appropriate parametric statistical tests. For data that did not fall within equal variance-Gaussian distribution, we performed appropriate nonparametric statistical tests. For all experiments, *P < 0.05, **P < 0.01, ***P < 0.001.

## Supplementary information

Supplementary information

## Data Availability

The RNA-sequencing data were deposited in Sequence Read Archive and the accession number is PRJNA514703.
