# Supplementary material for: MEF2C regulates osteoclastogenesis and pathologic bone resorption via c-FOS
Source: Bone Res. 2021 Jan 11;9:4. doi: 10.1038/s41413-020-00120-2 (PMC7797478; doi:10.1038/s41413-020-00120-2)
Supplement: Supplementary file 1 — Supplementary information [file 41413_2020_120_MOESM1_ESM.docx]

**Supplemental Fig. 1. MEF2C expression in human osteoclast precursor cells**

**a-j** Human osteoclast precursor cells (OCPs) were cultured with M-CSF (20ng/ml) and RANKL (40ng/ml) for the indicated times. **a** RT-PCR analysis of *MEF2C* mRNA normalized relative to *TBP* mRNA. **b** Immunoblot of whole cell lysates with MEF2C and p38 antibodies. p38 was used as a control. **c** Human osteoclast precursor cells were nucleofected with control or MEF2C siRNAs. Immunofluorescence staining of MEF2C in OCPs that were cultured with M-CSF. Representative images from 3 independent experiments are shown. Right panels: enlarged images corresponding to white boxes in left panels. Scale bar, 100μm. **d** Enlarged images of control conditions in Fig.1c showing human multinuclear TRAP-positive osteoclasts. **e-j** Human osteoclast precursor cells were transduced with adenoviral particles encoding GFP or MEF2C-FLAG. **e** RT-qPCR analysis of mRNA of MEF2C-FLAG fusion gene normalized relative to *TBP* mRNA. n=3. **f** Immunoblot of whole cell lysates with FLAG and MEF2C antibodies. p38 was used as a loading control. Arrow head, endogenous MEF2C. Arrow, exogenous MEF2C-FLAG. **g** Immunofluorescence staining of human MEF2C in OCPs transduced with GFP or MEF2C-FLAG. Enlarged images of white boxes are shown in the right panel. Scale bar, 100μm. **h** Quantitation of fluorescence intensity of MEF2C in OCPs. Percentage of cells with fluorescence intensity above threshold is shown n=4. **i**. Viability of MEF2C overexpressed cells was measured by XTT assay. n=6. **j** RT-qPCR analysis of *ITGB3*, *CTSK* and *CTR* mRNA after 96 hours of culture with or without RANKL (40 ng/ml) normalized relative to *TBP* mRNA (control samples without RANKL set at 1.0. n=7. Data are shown as mean ± SD. **p*<0.05, ***p*<0.01, ****p*<0.001.

**Supplemental Fig. 2. MEF2C expression in MEF2C^ΔMX^ mice.**

**a** Body weight and femur length of MEF2C^ΔMX^ mice and littermate control 16-week-old mice after induction of MEF2C deletion by Poly (I:C) at the age of 6 weeks. **b** Immunohistochemistry analysis of the distal femur stained for MEF2C. Upper panels: representative images from 4 independent experiments. Lower: bar graphs showing the quantitation of DAB-positive cells in chondrocytes, osteocytes/osteoblasts and bone marrow cells. n=4. Data are shown as mean ± SD. **p*<0.05. **c** Gating strategy of FACS-sorting of bone marrow cells. CD34, c-Kit and Ter119-postive cells were excluded from the downstream analysis. **d** Immunofluorescence staining of MEF2C in FACS-sorted cells (T cells (CD45^+^CD3^+^CD19^-^), B cells (CD45^+^CD19^+^CD3^-^), neutrophils (CD45^+^CD3^-^CD19^-^Ly6G^+^), monocytes (CD45^+^CD3^-^CD19^-^Ly6G^-^Ly6C^med^CD11b^high^) and OCPs(CD45^+^CD3^-^CD19^-^Ly6G^-^Ly6C^high^CD11b^low/med^)). Representative images from 2 biological replicates are shown. **e** Mouse OCPs from MEF2C^ΔMX^ mice or littermate control wild type mice were cultured with M-CSF and RANKL. Immunoblot of whole cell lysates with anti-MEF2C and α-tubulin antibodies. **f** Immunofluorescence staining of MEF2C in mouse OCPs. Representative images from 5 biological replicates. Enlarged images of white boxes are shown in the right panels. Scale bar, 100μm.

**Supplemental Fig. 3. RANKL signals were not regulated by MEF2C deficiency**

**a-c** Human osteoclast precursor cells (OCPs) were cultured with M-CSF (20ng/ml) and RANKL for the indicated times. **a** Immunoblot of RANK in OCPs with or without MEF2C KD. Representative images from 3 independent experiments. **b** Immunoblot of whole lysates with IkB, phospho-ERK, ERK, phospho-p38, and p38 antibodies. ERK and p38 were used as controls. **c** Immunoblot of nuclear lysates with p65, p50, LaminB1 and a-tubulin antibodies. **d** GO analysis showing significant enrichment of MEF2C-associated genes in OCPs/Macrophages.

**Supplemental Fig. 4. Fos expression in human OCPs.**

**a** Human osteoclast precursor cells were cultured with M-CSF (20ng/ml) and RANKL (40ng/ml). Immunoblot of nuclear lysates with c-FOS and LaminB1 antibodies. The images from two independent donors (upper panels: Donor 2 and lower panels: Donor 3).

**Supplemental Fig. 5. Forced expression of Fos in MEF2C^ΔMX^ cells.**

**a** UCSC Genome Browser tracks showing normalized tag density of MEF2C and PU.1 ChIP-seq and ATAC-seq in the upstream region of FOS. MEF2C data are from reference 30 and PU.1 and ATACseq data are from GSE97779 (reference 33). **b-f** Mouse osteoclast precursor cells (OCPs) were transduced with retroviral particles encoding GFP or Fos. **b** RT-qPCR analysis of *Fos* mRNA normalized relative to *Hprt* mRNA. WT; n=5, KO; n=6. **c** Immunoblot of whole cell lysates with NFATc1 and α-tubulin antibodies. α-tubulin was used as a control. **d** Densitometric quantitation of NFATC1 band intensity normalized to α-tubulin band intensity. n=3. **e** RT-PCR analysis of *Nfatc1* mRNA normalized relative to *Hprt* mRNA at 48 hours after RANKL stimulation. (WT; n=5, KO; n=6.) **f** RT-qPCR analysis of *Itgb3, Ctsk and Ctr* mRNA normalized relative to *Hprt* mRNA at 72 hours after RANKL stimulation. WT; n=5, KO; n=5. Data are shown as mean ± SD. **p*<0.05.

**Supplemental Fig. 6. NFATc1 expression in human osteoclast precursor cells.**

**a** Human OCPs were cultured with M-CSF (20ng/ml) and RANKL (40ng/ml). NFATc1 expression in RANKL-stimulated OCPs at the indicated times. Immunoblot of whole cell lysates with NFATc1 and p38 antibodies. p38 was used as a control. **b-d** Human osteoclast precursor cells were transduced with adenoviral particles encoding GFP or MEF2C-FLAG. **b** RT-qPCR analysis of *NFATC1* mRNA after 48 hours of RANKL stimulation normalized relative to *TBP* mRNA. Control samples without RANKL were set at 1.0. n= 7 **c** Immunoblot of nuclear lysates with NFATC1, LaminB1, and α-tubulin antibodies. **d** Densitometric quantitation of NFATC1 band intensity at 48-hour time point. (n=5). Data are shown as mean ± SD. **p*<0.05.

**Supplemental Fig. 7. MEF2C expression increases in synovial CD14+ cells from patients with rheumatoid arthritis.**

Signal intensity of *MEF2C*in synovial CD14+ cells from RA patients or control CD14+ cells from healthy donors. The microarray data for RA synovial CD14+ cells was obtained from GSE97779. n=5 in healthy donor, n=10 in RA. * p<0.05.
